# The Building Blocks of Antimicrobial Resistance in *Pseudomonas aeruginosa*: Implications for Current Resistance-Breaking Therapies

**DOI:** 10.3389/fcimb.2021.665759

**Published:** 2021-04-16

**Authors:** R. Frèdi Langendonk, Daniel R. Neill, Joanne L. Fothergill

**Affiliations:** Institute of Infection, Veterinary and Ecological Science, University of Liverpool, Liverpool, United Kingdom

**Keywords:** multidrug-resistance (MDR), resistance mechanism interplay, antimicrobial resistance (AMR), resistance-breaking therapy, adjuvant therapies, *Pseudomonas aeruginosa*

## Abstract

*P. aeruginosa* is classified as a priority one pathogen by the World Health Organisation, and new drugs are urgently needed, due to the emergence of multidrug-resistant (MDR) strains. Antimicrobial-resistant nosocomial pathogens such as *P. aeruginosa* pose unwavering and increasing threats. Antimicrobial stewardship has been a challenge during the COVID-19 pandemic, with a majority of those hospitalized with SARS-CoV2 infection given antibiotics as a safeguard against secondary bacterial infection. This increased usage, along with increased handling of sanitizers and disinfectants globally, may further accelerate the development and spread of cross-resistance to antibiotics. In addition, *P. aeruginosa* is the primary causative agent of morbidity and mortality in people with the life-shortening genetic disease cystic fibrosis (CF). Prolonged periods of selective pressure, associated with extended antibiotic treatment and the actions of host immune effectors, results in widespread adaptive and acquired resistance in *P. aeruginosa* found colonizing the lungs of people with CF. This review discusses the arsenal of resistance mechanisms utilized by *P. aeruginosa*, how these operate under high-stress environments such as the CF lung and how their interconnectedness can result in resistance to multiple antibiotic classes. Intrinsic, adaptive and acquired resistance mechanisms will be described, with a focus on how each layer of resistance can serve as a building block, contributing to multi-tiered resistance to antimicrobial activity. Recent progress in the development of anti-resistance adjuvant therapies, targeting one or more of these building blocks, should lead to novel strategies for combatting multidrug resistant *P. aeruginosa.* Anti-resistance adjuvant therapy holds great promise, not least because resistance against such therapeutics is predicted to be rare. The non-bactericidal nature of anti-resistance adjuvants reduce the selective pressures that drive resistance. Anti-resistance adjuvant therapy may also be advantageous in facilitating efficacious use of traditional antimicrobials, through enhanced penetration of the antibiotic into the bacterial cell. Promising anti-resistance adjuvant therapeutics and targets will be described, and key remaining challenges highlighted. As antimicrobial stewardship becomes more challenging in an era of emerging and re-emerging infectious diseases and global conflict, innovation in antibiotic adjuvant therapy can play an important role in extending the shelf-life of our existing antimicrobial therapeutic agents.

## *P. aeruginosa* the Pathogen

*P. aeruginosa* is a ubiquitous environmental bacterium capable of establishing opportunistic infections in both plants and animals, including humans (Mathee et al., 2008). It is the primary cause of Gram-negative nosocomial infections and of lung infections in people with cystic fibrosis (CF) (Pendleton et al., 2013) (WHO publishes list of bacteria for which new antibiotics are urgently needed), chronic obstructive pulmonary disease (COPD) and non-CF bronchiectasis (Streeter and Katouli, 2016). *P. aeruginosa* has a large genome of 5.5-7 million base pairs, with remarkable plasticity (Abril et al., 2019; Stover et al., 2000). Its ability to adapt to a range of environmental niches and its high nutritional versatility stems from this genome plasticity. In addition, a large variety of intrinsic and acquired resistance mechanisms exist within the *P. aeruginosa* population (El Zowalaty et al., 2015). Understanding these mechanisms of resistance and their interplay can help develop targeted therapies for combatting MDR *P. aeruginosa* infections.

## Resistance in *P. aeruginosa*

Antibiotic resistance mechanisms can be broadly divided into three categories; intrinsic, acquired and adaptive. Intrinsic resistance mechanisms are those genetically encoded in the core genome of the organism, whereas adaptive resistance mechanisms are those induced by environmental stimuli, and acquired resistance arises from gain of resistance genes from other organisms or as a consequence of selection of advantageous mutations (Tenover, 2006).

Intrinsic resistance mechanisms of *P. aeruginosa* include its low outer membrane permeability (12- to 100- fold lower than that of *Escherichia coli*), the presence of antibiotic efflux pumps and β-lactamases, such as OXA-50 and AmpC (Bellido et al., 1992; Slama, 2008; Lister et al., 2009; Szczepanowski et al., 2009). Acquired resistance mechanisms from horizontal gene transfer include acquisition of transferable aminoglycoside modifying enzymes and β-lactamases, while acquired resistance as a result of *de novo* mutational events often takes the form of overexpression of efflux pumps and β-lactamases, along with decreased expression or modification of target sites and porins (Meletis and Bagkeri, 2013). Acquired resistance through adaptive mutations is common in CF isolates due to the prolonged periods of selective pressure during extended antibiotic treatment of chronic infection and the actions of host immune effectors (Oliver et al., 2000; Dettman et al., 2016). Adaptive resistance mechanisms are those that are induced through external stimuli, such as stress factors and the presence of certain antibiotics. This is different from acquired mutational resistance as adaptive resistance is transient and unstable. Adaptive resistance mechanisms are not permanent, unlike mutational events, and become inactive upon removal of the stress factor (Moradali et al., 2017). Adaptive resistance often involves regulatory pathways and leads to altered gene expression, changes in protein production or target alteration. For example, two-component signaling systems (TCSS), MexXY induction and biofilm formation (Hocquet et al., 2003; Fernández et al., 2010; Sun et al., 2018a; Coleman et al., 2020).

In this review, we will discuss the different resistance mechanisms *P. aeruginosa* employs to evade antibiotic action. Intrinsic resistance mechanisms, such as low-outer membrane permeability, efflux, lipopolysaccharide (LPS) modification and the bacterial enzyme AmpC, will be described, with a focus on how these contribute to high-level resistance *via* interplay with acquired mutations and adaptive mechanisms. In addition, we will outline the roles of bacterial enzymes and commonly acquired resistance mechanisms, as well as phenotypic contributions to resistance, such as motility and biofilm formation. Novel therapeutic efforts that directly target these resistance mechanisms will be reviewed and future priorities discussed.

## Surface Proteins and Systems: Porins

**Figure 1:**
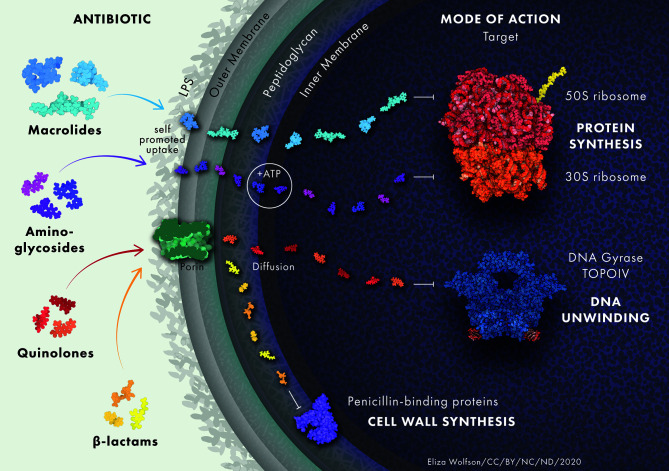
Antibiotic uptake in *P. aeruginosa*. Aminoglycosides diffuse through the outer membrane due to electrostatic interactions between the positively charged aminoglycoside and the negatively charged LPS. They undergo rapid energy-dependent accumulation into the cell with the use of electron transport and ATP hydrolysis. Once inside the cell, aminoglycosides bind to the 30S subunit of ribosomes to inhibit protein synthesis. β-lactam antibiotics target trans-peptidases on the cytoplasmic membrane that play a vital role in the assembly and cross-linking of cell wall peptidoglycan. Macrolides diffuse across the lipid bilayer due to their hydrophobic nature. They bind to the 50S ribosomal subunit and cause the dissociation of peptidyl-tRNA from the ribosome inside the cell. Quinolones inhibit DNA gyrase and DNA topoisomerase IV in Gram-negative and Gram-positive bacteria, respectively, leading to double stranded DNA breaks. Protein and drug structures were generated with Protein Imager (Tomasello et al., 2020).

Antibiotics must cross the bacterial membrane in order to act on intracellular targets (**Figure 1**) (Tipper, 1985; Tenson et al., 2003; Jacoby, 2005; Shakil et al., 2008). Accumulation of antibiotics in the cell is intrinsically diminished in *P. aeruginosa* due to its low outer membrane permeability (Lambert, 2002). The relative dearth of porins within the outer membrane (OM) of *P. aeruginosa* decreases the rate at which antibiotics can penetrate the cell (Trias et al., 1989). Porins not only function in transporting nutrients and other molecules across the membrane, but they also play a role in signaling, adhesion and stability of the membrane (Achouak et al., 2006). P*. aeruginosa* has 26 different types of porin, of which OprF is the most abundant in *P. aeruginosa* and has been implicated in a variety of functions (Chevalier et al., 2017).

### OprF – A Major Porin With a Key Role in Lung Infections

OprF is present in two forms, the highly abundant two-domain closed channel and the single domain open channel. The open channel conformation occurs in <5% of the OprF protein population (Sugawara et al., 2012). The two-domain closed channel conformation is thought to interact with peptidoglycan and therefore to play a structural role by stabilizing the OM (Hancock et al., 1981; Woodruff and Hancock, 1989). The single domain open channel spontaneously transitions between different sub-conformations that are weakly conductive and exists for only a short time in its fully open state. The low occurrence of the open channel and its weak conductive state aid in the low permeability of *P. aeruginosa* (Nestorovich et al., 2006). Clinical OprF deficient mutants of *P. aeruginosa* with increased antibiotic resistance have been observed (Piddock et al., 1992). However, several studies have shown that complete loss of OprF alone does not seem to be a major route to antibiotic resistance amongst clinical isolates (Pumbwe et al., 1996; Brinkman et al., 1999; Pumbwe and Piddock, 2000). The direct role of OprF in antibiotic resistance is somewhat ambiguous, despite being implicated in quorum sensing and biofilm formation (Chevalier et al., 2017). Interestingly, OprF is detectable in CF patient sputum samples and is upregulated 40-fold in anaerobic conditions, in comparison to aerobic conditions. It has been found to be necessary for growth in the anaerobic environment of CF sputum (Yoon et al., 2002). Overexpression of OprF was also observed in a *P. aeruginosa* artificial sputum biofilm model of the CF lung and was shown to be necessary for the tight microcolony formation that occurs during the early stages of biofilm formation in the CF lung (Sriramulu et al., 2005). Therefore, although the direct role of OprF in antimicrobial resistance may be ambiguous, there is evidence of a wider role in the establishment of other *P. aeruginosa* resistance mechanisms during lung infections.

### OprD – Enhancing Resistance to Carbapenems and β-lactams

OprD is the second major porin protein in *P. aeruginosa* and one of the most well-characterized, due to its involvement in the entry of carbapenem antibiotics, mainly imipenem and meropenem (Triass and Nikaido, 1990; Yoneyama et al., 1995). Carbapenem resistance has been linked to mutations causing downregulation of *oprD* expression and to OprD amino acid substitution mutations found in hypermutator isolates (Richardot et al., 2015).

OprD has been found in high abundance in biofilm outer membrane vesicles (OMVs) (Park et al., 2015). OMVs bud from the outer-membrane and are filled with periplasmic components. They have several functions that serve to enhance bacterial survival, such as modulating host immunity, delivering virulence factors, acquiring nutrients and contributing to structural support in biofilms (Schwechheimer and Kuehn, 2015). OprD-rich OMVs are thought to absorb carbapenems through OprD, leading to accumulation in the OMV and simultaneously lowering the carbapenem concentration in the bacterial cell (Chevalier et al., 2017). Similarly, *P. aeruginosa* may package OprD into OMVs to decrease cellular levels of this porin and thereby reduce the passage of carbapenems across the membrane and into the cell (Park et al., 2015).

Chronic *P. aeruginosa* infection in CF can lead to reduced oxygen tension, due to nearby polymorphonuclear leukocyte activity, causing some bacteria to grow anaerobically (Hassett et al., 2009; Kolpen et al., 2017). Increased antibiotic tolerance is observed in *P. aeruginosa* that grow in well established, anoxic biofilms (Hill et al., 2005). Several studies have found strongly decreased expression of *oprD* in 48-hour and 96-hour anoxic biofilms, which may partly explain the enhanced resistance to carbapenems in biofilm (Dötsch et al., 2012; Tata et al., 2016). This is in contrast to *oprF*, which is upregulated under anoxic environments such as the CF lung (Bukhari and Aleanizy, 2020). Increased expression of *oprF* would not substantially increase membrane permeability, as the closed channel is the most common form of OprF. The increase in closed channel OprF porins along with the decrease in *oprD* expression may work in conjunction to further lower outer membrane permeability in *P. aeruginosa* and protect the cell from antibiotic permeation, although this has not been experimentally determined. Alternatively, the benefit derived from increased expression of *oprF* in the CF lung may relate to its role in QS signaling and/or as a host immune system sensor (Fito-Boncompte et al., 2011).

Khuata et al. found that sialylation occurs in multiple porin proteins in the presence of normal human serum. OprD was identified as a sialoglycoprotein and sialylation of OprD resulted in a decreased interaction with β-lactam antibiotics in comparison to non-sialylated OprD. It was also found that piperacillin and ceftazidime were able to penetrate non-sialylated OprD much more readily than the sialylated form. Accordingly, in the presence of normal human serum, increased survival in the presence of these antibiotics is observed, as a result of OprD sialylation (Khatua et al., 2014). These observations highlight the effect that mode of growth and host factors can have on the susceptibility of *P. aeruginosa* to antibiotics.

### OprH – The Small Porin

OprH is a 21.6 kDa porin protein and is the smallest porin found in *P. aeruginosa* (Kucharska et al., 2016). OprH interacts with LPS through electrostatic interactions and is implicated in polymyxin resistance through the low Mg^2+^ concentration sensing PhoPQ and PmrAB TCSS (Edrington et al., 2011; Kucharska et al., 2016). The genes for the PhoPQ TCSS form an operon with *oprH*. However, studies with *oprH* knockout *P. aeruginosa* have demonstrated that the porin is not essential for polymyxin resistance. Instead, OprH is thought to play a supplementary role in polymyxin resistance through stabilizing the OM during Mg^2+^ starvation (Macfarlene et al., 2000). OprH has also been linked with gentamicin resistance in small colony variants of *P. aeruginosa*, although it is not clear whether upregulation of the operon alone was responsible for the resistance phenotype, as several other genes were also upregulated (Wei et al., 2011).

## Surface Proteins and Systems: Efflux Pumps

**Figure 2:**
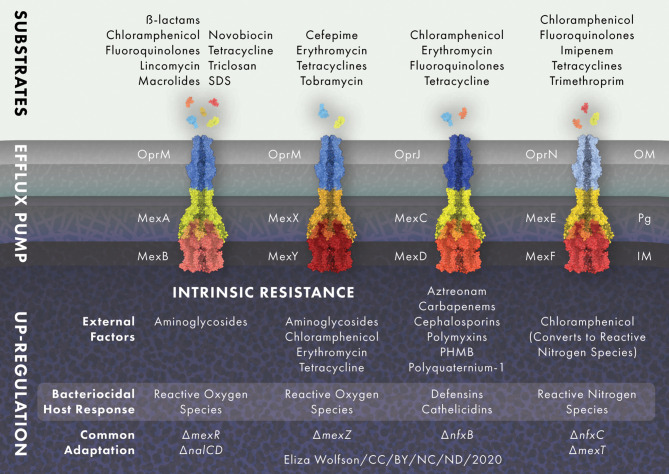
RND efflux pump expression is modulated in response to external stress. MexAB-OprM and MexXY-OprM are positively regulated by reactive oxygen species (ROS) and MexEF-OprN is induced by reactive nitrogen species (NOS), whilst MexCD-OprJ responds to membrane damaging agents and MexXY-OprM to ribosome blocking substances such as chloramphenicol, erythromycin, tetracycline and aminoglycoside antibiotics. Common adaptations leading to up-regulation of efflux pumps include; loss of active transcriptional regulators *mexR, nalC* and *nalD* for MexAB-OprM, loss of the transcriptional repressor *mexZ* for MexXY-OprM, loss of transcriptional regulator *nfxB* for the MexCD-OprJ efflux system and *nfxC* type mutations and/or loss of active transcriptional activator *mexT* for MexEF-OprN. Protein and drug structures were generated with Protein Imager (Tomasello et al., 2020).

Bacterial efflux pumps are categorized into five families, the two ancient super families that are made up of the adenosine triphosphate binding cassette (ABC) and the major facilitator super family (MFS), and three smaller families that consist of the multidrug and toxic compound extrusion family (MATE), the small MDR family (SMR) and the resistance nodulation-cell division family (RND) (Marquez, 2005). RND efflux pumps are the main multidrug efflux systems responsible for antibiotic extrusion and resistance in *P. aeruginosa* (Poole, 2001).* P. aeruginosa* possesses at least 12 RND efflux pumps, of which four are of clinical relevance and are the main contributors to antibiotic resistance through efflux (Stover et al., 2000; Alcalde-Rico et al., 2018), namely, MexAB-OprM, MexCD-OprJ, MexEF-OprN and MexXY-OprM (Dreier and Ruggerone, 2015) (**Figure 2**). All RND efflux pumps cross the cytoplasmic membrane, the periplasm and the OM (Nikaido, 2010). The RND efflux pump complex is comprised of the secondary active efflux pump protein embedded in the inner membrane, the outer membrane factor protein, and a membrane fusion protein that extends through the periplasm and connects the two (Daury et al., 2016; Hernando-Amado et al., 2016). Efflux pumps are powered by proton motor force and are able to export drugs from both the periplasm and the cytosol into the environment (Paulsen, 2003; Poole, 2008). Efflux and the low-permeability OM work in a cooperative manner, whereby those antibiotics that manage to permeate the OM are quickly extruded by efflux pumps, back into the environment (Nikaido, 2010).

Efflux pumps not only function in antimicrobial efflux, but are also recruited in response to cellular stress. Stress response signals including host factors, detergents and other endogenous inducers of the bacterial stress response may function as pressures for the selection of antimicrobial resistant efflux-expressing mutants, even in the absence of antimicrobials (Poole, 2014; Dreier and Ruggerone, 2015). For example, inflammation in the CF lung means *P. aeruginosa* is continuously exposed to reactive oxygen species (ROS), which is likely to drive the high prevalence of MexAB-OprM and MexXY-OprM over-expressing mutants (Poole, 2005) Additionally, *in vitro* studies have shown that ROS exposure selects for MexXY-OprM expressing, aminoglycoside resistant mutants (Fraud et al., 2008).

### MexAB-OprM – The Constitutively Active Pump

The MexAB-OprM efflux pump is constitutively active in *P. aeruginosa* wild-type strains and, along with other RND efflux systems, causes low level resistance but can also significantly contribute to the development of multi-drug resistance (Goli et al., 2016). MexAB-OprM extrudes carbapenems, chloramphenicol, fluoroquinolones, lincomycin, macrolides, novobiocin, penicillins, tetracyclines and all β-lactams (except imipenem and biapenem), as well as the antiseptic triclosan and the surfactant sodium dodecyl sulfate. MexAB-OprM null mutants are markedly susceptible to all of the aforementioned antimicrobials, whilst mutants that overproduce MexAB-OprM show high levels of resistance (Masuda and Ohya, 1992; Li et al., 1995; Srikumar et al., 1998; Köhler et al., 1999; Chen et al., 2016). MexAB-OprM consists of an inner membrane protein, MexA, and a fusion protein, MexB, that links MexA to the outer membrane protein OprM (Nehme et al., 2004). The efflux pump proteins are encoded in an operon, the expression of which is regulated by MexR (Poole et al., 1996a; Andrésen et al., 2010). MexR is encoded upstream of the *mexAB-oprM* operon and acts as a transcriptional repressor by binding to the *mexA-mexR* intergenic region near the promotors of *mexR* and a secondary promotor for *mexA* (Poole et al., 1996a; Evans et al., 2001). Loss of a functioning MexR causes hyperexpression of *mexAB-oprM.* Mutations in *mexR* found in clinical *P. aeruginosa* isolates can lead to the expression of MexR protein that is no longer able to dimerize or bind to DNA to repress *mexAB-oprM* expression. In addition, mutations leading to premature termination of the MexR peptide resulting in complete absence of the production of a functioning MexR have been identified in clinical isolates (Choudhury et al., 2016; Pan et al., 2016).

### MexCD-OprJ – Expressed in *nfxB* Mutants

MexCD-OprJ is not normally expressed in wild-type strains and is functional in *nfxB* mutants only, it is therefore not classified as contributing to the natural resistance of *P. aeruginosa* (Poole et al., 1996b; Morita et al., 2001). Antibiotic substrates of the MexCD-OprJ efflux system are chloramphenicol, erythromycin, fluoroquinolones and tetracycline. The NfxB protein negatively regulates MexCD-OprJ and different mutations within the *nfxB* gene lead to varying levels of MexCD-OprJ expression, leading to clinical isolates with different levels of resistance to antimicrobials (Masuda et al., 1996).

### MexEF-OprN - The Positively Regulated Pump

The MexEF-OprN efflux system extrudes chloramphenicol, fluoroquinolones, tetracycline, trimethoprim and imipenem (Maseda et al., 2000; Sobel et al., 2005). The *mexEF-oprN* operon is regulated by the transcriptional activator MexT (Köhler et al., 1999). MexT positively regulates *mexEF-oprN* expression, in contrast to most other RND-efflux systems, which are negatively regulated by a repressor protein (Poole, 2005). However, often wild-type strains of *P. aeruginosa* carry an inert *mexT* gene encoding inactive MexT, causing the suppression of *mexEF-oprN* expression (Maseda et al., 2000). Besides acting as a positive regulator for the expression of the *mexEF-oprN* efflux system when MexT is active, it also acts as a repressor of *oprD* transcription. Active MexT leads to a reduction of the OprD porin in the bacterial membrane, causing increased resistance to carbapenem antibiotics (Ochs et al., 1999). The multidrug resistant *nfxC*-type mutant overexpresses the *mexEF-oprN* operon and is resistant to chloramphenicol, fluoroquinolones, tetracycline, trimethoprim and imipenem (Köhler et al., 1997).

### MexXY-OprM – The Major Contributor to Aminoglycoside Resistance

The MexXY-OprM efflux system contributes to *P. aeruginosa* intrinsic resistance to aminoglycosides, tetracycline, erythromycin and cefepime (Masuda et al., 2000; Hocquet et al., 2006). MexXY-OprM is regulated by the MexZ repressor protein and mutations in or around *mexZ* lead to overproduction of MexXY-OprM (Llanes et al., 2004; Vogne et al., 2004). The *mexZ* gene is one of the most commonly mutated in isolates from people with CF, leading to loss of MexXY-OprM repression and thereby increasing its expression (Smith et al., 2006; Feliziani et al., 2010). Mutations in *mexZ* that arise during long-term infection in CF can result in resistance to tobramycin, a first-line antibiotic in CF clinics (Marvig et al., 2015; Prickett et al., 2017). One study looking at levels of *mexY* expression and *mexZ* mutation found that both were present at a significantly higher frequency in adults with chronic infection than in children with chronic or new infections and that *mexZ* mutation strongly correlated with aminoglycoside resistance (Prickett et al., 2017). This suggest that *mexZ* mutation undergoes positive selection during long term chronic infection.

## LPS Modification

LPS is a central component of the Gram negative bacterial membrane. It serves as the outer leaflet of the OM and is essential for host-pathogen interactions, virulence and antimicrobial resistance (Lam et al., 2011). P*. aeruginosa* LPS is composed of three domains: the core oligosaccharide, the lipid A portion and the O-antigen region (Gellatly and Hancock, 2013). Modification of LPS is a resistance mechanism that protects the bacterial cell against cationic antimicrobial peptides (CAPs) and polymyxin antibiotics (Baron et al., 2016). LPS modification in response to a low Mg2^+^ environment, or to exposure to CAPs and polymyxins, is achieved by the addition of a 4-amino-4-deoxy-L-arabinose (L-Ara4N) moiety to the phosphate unit on the lipid A group of the LPS. Modification of the lipid A arabinose subunit leads to a lower net negative charge of the bacterial membrane, which leads to a decrease in electrostatic interactions with positively charged antimicrobials. The decrease in electrostatic interaction between the antibiotic and the membrane prevents the drug from binding the LPS which would normally destabilize and disrupt the membrane (Falagas et al., 2010). LPS modification in *P. aeruginosa* is regulated by several two-component signaling systems (TCSS): PmrAB, PhoPQ, CprRS and ParRS (Fernández et al., 2010; Fernández et al., 2012). These TCSS regulate the transcription of the *arnBCADTEF* operon, which encodes the genes responsible for the addition of L-Ara4N to the lipid A group, in response to a variety of environmental signals (Skiada et al., 2011). PmrAB and PhoPQ regulate the activation of *arnBCADTEF* under Mg2^+^ deficient growth conditions (McPhee et al., 2003). ParRS and CprRS both play an active part in adaptive resistance against CAPs and can stimulate *arnBCADTEF* upregulation, independently of each other, in the presence of these peptides (Fernández et al., 2010; Fernández et al., 2012). Mutations in these regulatory TCSS that lead to the constitutive activity of the *arn* operon confer colistin resistance to *P. aeruginosa* (Sciuto and Imperi, 2018). One study found that mutations in the *pmrB* histidine kinase gene of the PmrAB TCSS commonly arise during the development of high level polymyxin resistance in experimental evolution experiments performed with CF isolates (Moskowitz et al., 2012; Jochumsen et al., 2016). Conversely, a missense mutation in *pmrB* in isolates taken from a murine model of *P. aeruginosa* chronic respiratory infection has been found to be associated with increased susceptibility to a wide range of antibiotic classes but also with resistance to the host antimicrobial enzyme lysozyme (Fothergill et al., 2014; Bricio-Moreno et al., 2018). High level resistance to polymyxin antibiotics is forged through epistatic interactions between multiple regulatory genes and can occur *via* several pathways (Jochumsen et al., 2016), but a functional *arn* operon and the aminoarabinosylation of the lipid A are requirements for polymyxin and colistin resistance in *P. aeruginosa* (Sciuto and Imperi, 2018).

## Bacterial Enzymes

### β-lactamases

β-lactam antibiotics interfere with cell wall synthesis and disrupt peptidoglycan recycling by inhibiting penicillin binding proteins (PBP), enzymes responsible for synthesis and maintenance of peptidoglycan (Sauvage et al., 2008; Johnson et al., 2013). β-lactamases are enzymes that interfere with this process by hydrolyzing the β-lactam ring that is present in all β-lactam antibiotics (Santajit and Indrawattana, 2016). β-lactamases are classified according to different systems, the Ambler classification groups enzymes according to amino acid sequence and the Bush-Jacoby-Medeiros system classifies enzymes according to function and phenotype (Bush and Jacoby, 2010).

#### AmpC - The Chromosomally Encoded β-lactamase

The β-lactamase AmpC is an Ambler class C antibiotic inactivating enzyme. It contributes to the natural resistance of *P. aeruginosa* and is constitutively produced at basal levels (Juan et al., 2005). The chromosomal *bla_AmpC_*gene is drug inducible with aminopenicillins and cephalosporins, whereupon AmpC inactivates the β-lactam antibiotic through the process of hydrolysis (Masuda et al., 1999). AmpC induction occurs through two different mechanisms: perturbation of the peptidoglycan recycling pathway and through loss of PBP4 (Cavallari et al., 2013). Peptidoglycan is composed of cross-linked, alternating *N*-acetylglucosamine (GlcNAc) and *N*-acetylmuramic acid (MurNAc) residues (Vollmer et al., 2008). Peptidoglycan recycling requires the collaboration of PBPs, endopeptidases, carboxypeptidases, *N*-acetylmuramoyl-l-alanine amidases and lytic transglycosylases (LTs) (Johnson et al., 2013). LT’s cleave the β-1,4 glycosidic linkage between GlcNAc and MurNAc residues during growth and division, to generate new material to be inserted into the peptidoglycan layer. This cleavage causes the release of anhydromuropeptides (anhMPs) (Lee et al., 2013). The anhMPs are transported to the cytoplasm through an inner membrane permease AmpG, where they are processed for recycling back into the peptidoglycan biosynthesis pathway by the β-*N*-acetylglucosaminidase NagZ and the *N*-acetylmuramoyl-l-alanine amidase AmpD. Disruption of anhMP processing leads to their accumulation in the cytoplasm where they can bind to AmpR, the transcriptional regulator of AmpC. Two anhMPs, *N*-acetylglucosamine-1,6-anhydro-*N*-acetylmuramyl pentapeptide and 1,6-anhydro-*N*-acetylmuramyl pentapeptide, have been identified as signaling molecules that activate β-lactamase expression in *P. aeruginosa* (Lee et al., 2016).

Often β-lactamase resistance in clinical *P. aeruginosa* strains is caused by inactivation of the PBP4 encoding gene *dacB*, causing *ampC* overexpression and activation of the CreBC (BlrAB) TCSS (Moya et al., 2009). PBP4 functions to create an anhydromuropeptide (anhMP) that controls AmpR. Loss of PBP4 leads to the disappearance of this specific anhMP from the peptidoglycan recycling pathway, which changes AmpR regulation, thereby inducing *ampC* expression (Johnson et al., 2013). Upregulation of *ampC* is commonly observed amongst *P. aeruginosa* isolates from people with CF, it is selected for by clinical use of β-lactam antibiotics and the presence of hypermutable strains in the CF lung can hasten the emergence of this mutational overexpression (Mark et al., 2011; Llanes et al., 2013; López-Causapé et al., 2013; Aghazadeh et al., 2014).

#### Metallo β-lactamases

Metallo β-lactamases (MBLs) belong to Ambler classification group B, they contain zinc in the active site, as opposed to a serine based hydrolytic system (Queenan and Bush, 2007). The most clinically relevant MBLs in *P. aeruginosa* are the Verona integron-encoded MBLs (VIM) and active-on-imipenem type MBLs (IMP). They are often located on integrons along with other resistance genes, which allows these genes to integrate into the chromosome or a plasmid and disseminate through bacterial populations (Palzkill, 2013). The presence of MBL causes high-level resistance against carbapenem antibiotics (Feng et al., 2017).

#### Clinically Relevant ESBL

Extended spectrum β-lactamases (ESBL) hydrolyze oxyimino-aminothiazolyl cephalosporins (cefotaxime, cefuroxime, cefepime, ceftriaxone and ceftazidine) as well as penicillins and other cephalosporins, excluding cephamycins (Livermore and Brown, 2001). The most common ESBL in *P. aeruginosa* are the Ambler class A SHV-, VEB-, PER- and GES-type enzymes and the OXA-type enzymes from Ambler class D (Ortiz de la Rosa, et al., 2019). The presence of ESBLs confers a higher level of resistance to extended spectrum cephalosporins than AmpC, impermeability and efflux pump hyper-expression combined (Jiang et al., 2006). However, ESBL enzymes can be inhibited by clavulanic acid, sulbactam and tazobactam (Paterson and Bonomo, 2005). ESBL producing *P. aeruginosa* have been frequently isolated, although their detection is complicated by the activity of chromosomally encoded β-lactamases in *P. aeruginosa* (Livermore and Brown, 2001; Laudy et al., 2017).

### Aminoglycoside Modifying Enzymes

Aminoglycoside modifying enzymes (AME) are capable of catalyzing the modification of the -OH or -NH2 groups of the 2-deoxystreptamine nucleus or the sugar moieties of aminoglycoside antibiotics (Ramirez and Tolmasky, 2010). There are three types of AMEs; acetyltransferases (AACs), nucleotidyltransferases (ANTs) and phosphorlyl transferases (APHs). All of these decrease the binding affinity of the antibiotic to its target by transferring a functional group onto the aminoglycoside (Azucena and Mobashery, 2001). All three types of AMEs have been observed in *P. aeruginosa* and are common determinants of aminoglycoside resistance in non-CF isolates (Poole, 2011). AMEs are not, however, the most common aminoglycoside resistance mechanism in people with CF. Instead, impermeability and efflux are the more commonly observed events (Islam et al., 2009). However, studies have observed a variable prevalence of these enzymes in between 5.6% and 52% of isolates (MacLeod et al., 2000; Aghazadeh et al., 2013).

#### 16S rRNA Methylase

16S rRNA methylases are another group of modifying enzymes carried by *P. aeruginosa*. These enzymes are carried on plasmids and are thought to have come from an aminoglycoside producing organism that utilizes these enzymes to protect its own ribosomes from aminoglycoside action (Doi and Arakawa, 2007). 16S rRNA methylases cause the methylation of a specific nucleotide of the 16s rRNA A-site, to protect the ribosome from aminoglycoside activity (Doi and Arakawa, 2007). Strains carrying 16s rRNA methylase genes show a high level of resistance to most clinically relevant aminoglycoside antibiotics. The prevalence of these enzymes is not common in CF and other respiratory associated infection isolates (Yokoyama et al., 2003; Tada et al., 2013; Francisco et al., 2015) However, *rmtD* carrying *P. aeruginosa* have been reported in Africa, Asia, Europe and South America and are, along with ESBLs, often associated with a pan-resistant genotype (Fontes et al., 2011; Urbanowicz et al., 2019).

### DNA gyrase and topoisomerase IV

DNA gyrase and topoisomerase IV are both involved in DNA replication and management of chromosome integrity (Drlica and Zhao, 1997). DNA gyrase functions to cleave double stranded DNA, introduce negative supercoiling and re-ligate the strand in an ATP-dependent reaction (Levine et al., 1998). Topoisomerase IV functions by decatenating and relaxing positively supercoiled DNA (Corbett et al., 2005). Fluoroquinolones like ciprofloxacin act on DNA gyrase and topoisomerase IV to prevent the re-ligation step, leaving the DNA cleaved and thereby acting as a bacteriostatic antibiotic, blocking DNA replication (Drlica, 1999).

Resistance against ciprofloxacin occurs most commonly through mutations in the DNA gyrase subunits GyrA and GyrB or the topoisomerase subunits ParC/ParD but also through the overexpression of efflux (Yang et al., 2015). Mutations in *gyrA* and *gyrB* are commonly identified in CF associated infections and are likely to arise during prolonged antibiotic treatment with ciprofloxacin (Fothergill et al., 2010; Marvig et al., 2015). The most frequently observed mutations in both CF and non-CF isolates, as well as in *in vitro* experiments, are the substitution of an amino acid at position 83 in GyrA from threonine to isoleucine and an amino acid substitution of serine to leucine at position 87 in ParC (Higgins et al., 2003; Lee et al., 2005; Bruchmann et al., 2013). The second most common mutation is GyrA position 87, where tyrosine is substituted by asparagine, glycine or tyrosine (Wydmuch et al., 2005). These positions of GyrA are important for the binding of ciprofloxacin to DNA gyrase and the amino acid substitutions greatly lower the affinity of this binding (Willmott and Maxwell, 1993; Aldred et al., 2014). Resistance to ciprofloxacin is higher when both mutations in *gyrA* and *parC* are present, in comparison to only *gyrA* mutation (Pasca et al., 2012). However, it is thought that *gyrA* mutation is necessary for high level resistance against ciprofloxacin, due to the strong affinity of the native form of the protein for this antimicrobial. When only *gyrB, parC* or *parD* mutations are present or when efflux is the main mechanism of ciprofloxacin resistance, only low level resistance is observed (Rehman et al., 2019). A combination of efflux, *gyrAB* and *parCD* mutation leads to a higher level of resistance and again highlights that resistance in *P. aeruginosa* is complex, often involving multiple mechanisms (Dunham et al., 2010; Bruchmann et al., 2013).

### *fusA1* Mutation

Mutations arising in the *fusA1* gene lead to amino acid substitutions in a critical part of the translational machinery, elongation factor G (EF-G). Mutations within different domains of EF-G have been identified in clinical strains and are linked to decreased aminoglycoside susceptibility. Mutations in *fusA1* have been reported in both CF and non-CF isolates globally (Del Barrio-Tofiño et al., 2017; Rees et al., 2019). The mutation is often paired with upregulation of efflux, mainly the MexXY-OprM efflux pump, leading to strong aminoglycoside resistance. However, on its own, *fusA1* mutation can cause strains to be 4- to 8- fold more resistant to aminoglycosides. This was shown *in vitro*, where three *fusA1* mutants in domains II, III and IV were created (Bolard et al., 2017). The molecular mechanism linking mutations within EF-G to increased aminoglycoside resistance remains unclear. Aminoglycosides do not bind to or target EF-G, thus resistance to aminoglycosides must be an indirect effect of EF-G alteration (Gutierrez et al., 2013).

## Phenotypic Contributions to Resistance

### Swarming and Surfing

Bacterial motility is a vital part of *P. aeruginosa* pathogenicity and plays a role in host colonization and establishment of infection (Yeung et al., 2012). P*. aeruginosa* employs several different forms of motility that are environmentally dependent and often require the use of type IV pili, flagella or rhamnolipid surfactants (Overhage et al., 2007; Sun et al., 2018a; Sun et al., 2018b). RNA-seq and transposon mutant studies have shown that both swarming and surfing phenotypes are associated with significant changes in gene expression, leading to an increase in antibiotic resistance. Swarming motility was linked to the altered regulation of 1,581 genes, including 104 regulatory genes, including transcription factors, TCSS and sigma factors (Coleman et al., 2020). Surfing motility was linked to altered regulation of up to 2,078 genes and out of these, 31 resistome genes were identified which were known to cause an increase in antibiotic susceptibility when mutated (Sun et al., 2018a).

Both swarming and surfing play a role in adaptive resistance. They are environmentally dependent and associated with conferring a high level of antibiotic resistance in comparison to alternative motility phenotypes (Wang et al., 2013; Sun et al., 2018a; Coleman et al., 2020). Swarming cells have been shown to be significantly more resistant to aminoglycosides, β-lactams, chloramphenicol, ciprofloxacin, tetracycline, ethoprim, erythromycin and azithromycin (Coleman et al., 2020). Surfing cells however, showed significantly higher resistance to polymyxins, aminoglycosides, fluoroquinolones, tetracycline, chloramphenicol, trimethoprim and several β-lactams, but not to macrolides (Sun et al., 2018a). On semi-solid surfaces with low nitrogen concentrations, *P. aeruginosa* is known to utilize swarming motility. The conditions under which *P. aeruginosa* exhibits swarming motility appear to mimic those of the lung, where semi-solid mucous overlays epithelial cells. Likewise, the CF and bronchiectasis lungs contain high levels of mucin, the component necessary for surfing motility (Sun et al., 2018a). Although *P. aeruginosa* motility is often lost in long term chronic illness such as in CF, a plethora of genetic heterogeneity exists in the bacterial populations residing in the CF lung. Furthermore, swarming and surfing motility may occur during early colonization and adaptation to the CF lung.

### Biofilms

The biofilm mode of growth is a major impediment in the struggle to eradicate *P. aeruginosa* infection from the CF lung, due to the increased ability of bacteria in biofilms to withstand antibiotic treatment (Høiby et al., 2010). Biofilms are composed of bacteria surrounded by extracellular polymeric substances like exopolysaccharides, extracellular DNA and polypeptides (Rasamiravaka et al., 2015). This can lead to an increase in tolerance to antimicrobial agents of 100-1000 times, compared to planktonic cells (Ceri et al., 1999). Contributing features include quorum sensing, decreased ability to penetrate biofilm, presence of oxygen gradients, altered metabolism and slow bacterial growth rate (Olsen, 2015). Biofilms are common in chronic *P. aeruginosa* infection of the CF lung but have also been shown to be of significance in patients with COPD, bronchiectasis and chronic wounds (Costerton, 2001; Parameswaran and Sethi, 2012; Chalmers and Hill, 2013; Hassett et al., 2014; Rybtke et al., 2015). Airway mucins are found in abundance in people with CF, COPD and bronchiectasis. *In vitro* studies have shown that the presence of airway mucins are fundamental for the development of biofilm structures with enhanced tolerance to antimicrobials. Further, mucin may serve as a suitable attachment surface for *P. aeruginosa* biofilm formation (Landry et al., 2006; Müller et al., 2018).

Iglesias et al. investigated antibiotic pharmacodynamics within biofilm structures in the context of CF. Bacterial counts, metabolic activity and biomass of PAO1 biofilms grown in artificial sputum media (ASM) or trypticase soy-based medium were compared. They found that bacteria in ASM reached the same CFU and metabolic activity as biofilms formed in trypticase soy-based medium, although ASM biofilms grew slower and had a marginally higher biomass. When both biofilms were subjected to antibiotic treatment, ASM grown biofilms were substantially more resistant to tobramycin and colistin, but not ciprofloxacin and β-lactams (Diaz Iglesias and Van Bambeke, 2020). These studies indicate that environmental factors play an important role in determining biofilm structure and antimicrobial resistance. For comprehensive reviews on *P. aeruginosa* biofilms refer to: Olsen, 2015 and Maurice, Bedi and Sadikot, 2018 (Olsen, 2015; Maurice et al., 2018).

### Combinatorial Multidrug Resistance

**Figure 3:**
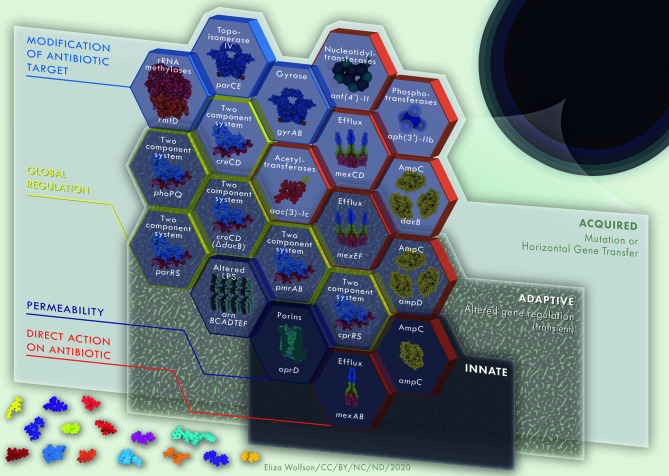
Multi-layered, interacting resistance mechanisms in *P. aeruginosa*. Innate (intrinsic) resistance mechanisms are encoded in the core genome of the organism, such as low outer membrane permeability, Mex-type efflux pumps and AmpC β-lactamase. Collectively, these comprise basal level resistance to antimicrobials, a foundation on which a variety of adaptive and acquired mechanisms of resistance may serve as building blocks to further enhance AMR in *P. aeruginosa*. Adaptive resistance mechanisms, including two-component regulatory systems, are environmentally dependent and will be expressed under certain conditions only. Mechanisms of resistance that are acquired, such as antibiotic modifying enzymes or mutations leading to antibiotic target modifications are strain dependent. The building blocks of innate, adaptive and acquired mechanisms of resistance contribute to a strong and multi-faceted protection against antimicrobial activity. Hexagon building blocks of resistance mechanisms are colored according to mechanism type; direct action on antibiotic, permeability, global regulation, modification of antibiotic target. Upper labels on hexagon building blocks describe resistance mechanism whilst lower labels define examples of each such systems. Protein and drug structures were generated with Protein Imager (Tomasello et al., 2020).

Multidrug resistance is often the result of the acquisition of external resistance genes and/or through mutational resistance (Diggle and Whiteley, 2020). Combining resistance genes encoding mechanisms previously described here, along with intrinsic resistance, can lead the development of MDR and XDR *P. aeruginosa.* MDR and XDR *P. aeru*ginosa arise due to the compilation of resistance mechanisms, amassing building blocks into a barrier of resistance strategies that protect the bacteria from antimicrobial assault. Intrinsic resistance forms the base layer of this barrier, with additional acquired and adaptive resistance mechanisms forming further layers, collectively constructing a robust barrier against antimicrobials (**Figure 3**). Antibiotic resistance genes can quickly and easily be disseminated through *P. aeruginosa* populations *en masse*, *via* megaplasmids (>420kb). Megaplasmids contain dynamic accessory genomes where frequent recombination and duplication events take place, leading to diverse and adaptive multidrug resistance traits (Cazares et al., 2020). Accordingly, resistance to antimicrobial agents in *P. aeruginosa* clinical isolates is highly complex, with frequent interplay between intrinsic, adaptive and acquired resistance mechanisms. AmpC, low outer membrane permeability and efflux systems often work together in resistance to carbapenems, chloramphenicol, fluoroquinolones, macrolides, penicillins, tetracyclines and β-lactams and resistance may be enhanced through the accumulation of mutations leading to up- or down- regulation of each of these systems. The outer membrane porin OprH works in conjunction with the two-component signaling systems PhoPQ and PmrAB in modifying the bacterial LPS to regulate protection to polymyxin antibiotics. Protection against polymyxin antibiotics is additionally mediated *via* TCSS ParRS and/or CprRS in the presence of cationic antimicrobial peptides. Aminoglycoside resistance is achieved through mechanisms such as aminoglycoside modifying enzymes, *fusA1* mutation, 16S rRNA methylation, along with MexXY-OprM upregulation. Likewise, *gyrA* and *parC* mutation leading to altered DNA gyrase and topoisomerase IV leads to fluoroquinolone resistance which can be heightened through the cooperation of MexAB-OprM and/or MexCD-OprJ, as well as MexEF-OprN. In addition, the presence of β-lactamases such as ESBLs and MBLs may further enhance carbapenem, cephalosporin and penicillin resistance. A perfect storm of highly resistant *P. aeruginosa* and a “dry pipeline” of traditional antimicrobials are driving innovation in novel therapeutic approaches that directly target resistance mechanisms. Counteracting these mechanisms could prolong the life of existing antimicrobials.

## Anti-Resistance Therapies for *P. aeruginosa*

### Outer Membrane Sensitizers

Increasing OM permeability to hydrophobic and amphiphilic compounds challenges the issue of intrinsic low-outer membrane permeability (Tümmler, 2019). For example, polymyxin B nonapeptide causes a 2- to 40-fold increase in susceptibility to ciprofloxacin, norfloxacin, and ofloxacin and 80- to 200-fold increase in susceptibility to nalidixic acid (Kubesch et al., 1987). Nalidixic acid is the precursor to ciprofloxacin, norfloxacin, and ofloxacin, and is not ordinarily more efficacious than the optimized antibiotics currently used in clinical practice. However, when the membrane becomes more permeable through polymyxin B nonapeptide treatment, nalidixic acid becomes a more powerful anti-pseudomonal (Tümmler, 2019). Polymyxin B nonapeptide is highly toxic and has therefore never been considered for clinical application (Tsubery et al., 2000). However, more than 30 years after the first report of OM sensitizers, three have been approved for clinical studies; the anti-protozoal pentamidine (Stokes et al., 2017) and the polymyxin B analogues SPR206 and SPR741 (Corbett et al., 2017; Vaara, 2019). None of these OM sensitizers, with the exception of SPR206, have been proven to have promising anti-pseudomonal activity. SPR206 is active against *P. aeruginosa* with a similar potency to polymyxin B and is currently undergoing phase-one clinical trial (Zhang et al., 2020). OM sensitizers appear to be a promising approach to resistance that can by-pass intrinsic, acquired and spontaneous resistance (Macnair and Brown, 2020). However, further investigation into additional non-toxic compounds that act on the *P. aeruginosa* OM is essential, especially for the treatment of polymyxin resistant isolates.

### Efflux Pump Inhibitors

#### Phe-Arg-β-naphthylamide (PAβN)

The most widely researched *P. aeruginosa* efflux-pump inhibiter (EPI) is Phe-Arg-β-naphthylamide (PAβN), a broad-spectrum peptidomimetic compound capable of interfering with all four clinically relevant *P. aeruginosa* RND efflux pumps. PAβN potentiates chloramphenicol, fluoroquinolones, macrolides, ketolides, oxazolidinones and rifampicin but not aminoglycosides or β-lactams (Lomovskaya and Bostian, 2006). The proposed mechanism of action for PAβN is that it functions as a substrate of the Mex-series efflux pumps and outcompetes the antibiotic for extrusion, preventing the antibiotic from leaving the cell (Mahmood et al., 2016). However, PAβN and derivatives of this compounds are not yet approved, as adverse toxicology and pharmacokinetic profiles were identified during phase 1 clinical trials (Renau et al., 2003). Instead, PAβN is solely utilized for research on EPI’s and AMR *in vitro*, rather than as a therapeutic, and may be used to validate the discovery of future EPI’s.

#### D13-9001

The pyridopyrimidine derivative D13-9001 is active against the MexAB-OprM efflux pump (Mahmood et al., 2016). D13-9001 has shown promising *in vitro* and *in vivo* activity, as well as high solubility and low-toxicity profiles (Yoshida et al., 2007). D13-9001 obstructs normal functioning of MexAB-OprM in two ways. Firstly, it prevents conformational changes by binding tightly to the hydrophobic trap. Secondly, it prevents substrate binding to MexB, through the interaction of the D13-9001 hydrophilic component and the substrate binding channel of MexB (Nakashima et al., 2013). EPIs will need to be broad-spectrum if they are to be used as an adjuvant to antibiotics that are substrates of several efflux-pumps. The specificity of D13-9001 would limit its usage to co-administration with antibiotics extruded exclusively by MexAB-OprM (Nakashima et al., 2013). However, a study by Ranjitkar et al. found that there are several mechanisms of resistance to D13-9001 potentiator activity in *P. aeruginosa*, when the agent is used together with carbenicillin, an antibiotic that is substrate specific to MexAB-OprM. Loss of potentiating activity of D13-9001 occurred rapidly due to a F628L substitution in *mexB*, which is known to play an important role in inhibitor binding (Ranjitkar et al., 2019).

#### Polyamine Derivatives

Polyamines are aliphatic carbon chains containing several amino groups and are essential organic polycations present in every form of life. Polyamines are implicated in cell maintenance and viability and in the functioning of a wide array of organ systems, including, the nervous and immune systems (Sánchez-Jiménez et al., 2019).

Fleeman et al. identified a polyamine scaffold as a strong efflux pump inhibitor with no direct antimicrobial activity. Five lead agents were found to potentiate aztreonam, chloramphenicol and tetracycline by causing a 5- to 8-fold decrease in the MIC90 (Fleeman et al., 2018). In addition, the polyamine derivatives did not disrupt the bacterial membrane, unlike other polyamines, which can lead to the identification of false positives for EPIs (Fleeman et al., 2018; Reza et al., 2019). Moreover, polyamines did not display toxicity to mammalian cell lines and did not inhibit calcium channel activity in human kidney cells (Fleeman et al., 2018).

#### Bacteriophage OMKO1

Phage therapy, the use of bacteriophages to infect and lyse bacterial cells, has been widely discussed (Chan et al., 2013; Gordillo Altamirano and Barr, 2019). Traditional phage therapy involves the administration of one, or a mixture, of phages that will invade the bacterial cell and clear infection (Waters et al., 2017). A different approach to phage therapy has been proposed, whereby phages would be used to steer antibiotic resistance evolution, selecting for phage resistance and antibiotic susceptibility. For example, the lytic *Myoviridae* bacteriophage, OMKO1, utilizes OprM of the multidrug efflux systems MexAB and MexXY as a receptor-binding site. Selection for resistance to OMKO1 bacteriophage attack creates an evolutionary trade-off in MDR *P. aeruginosa*, by changing the efflux pump mechanism, leading to an increased sensitivity to ciprofloxacin, tetracycline, ceftazidime and erythromycin, four drugs from different antibiotic classes (Chan et al., 2016). Phage steering can be achieved when the binding receptor for the bacteriophage is implicated in both antibiotic resistance and phage resistance. The advantage of this approach lies in the two distinct, and opposing, mechanisms leading to bacterial eradication (Gurney et al., 2020).

## Bacterial Enzyme Inhibitors

### β-lactamase Inhibitors

The prototypical example of successful anti-resistance therapeutics are the β-lactamase inhibitors. β-lactamase inhibitors such as clavulanic acid, sulbactam and tazobactam are widely used to combat resistance mediated by β-lactamases (Tooke et al., 2019). However, the majority of clinically used β-lactamase inhibitors have a limited spectrum and mainly target Ambler class A β-lactamases, excluding KPC-type β-lactamase. Progress has been made in the development of novel β-lactamase inhibitors with a wider spectrum of activity. Three novel β-lactamase inhibitors, avibactam, vaborbactam and relebactam, function against Ambler class A, C and D β-lactamases (Wong and Duin, 2017). However, only avibactam and relebactam are efficacious against *P. aeruginosa* infection (Aktaş et al., 2012; Barnes et al., 2018).

## DNA Gyrase and Topoisomerase IV Targeting Therapies

### Siderophore Mimic Bound DNA Gyrase B Inhibitors

Lamut et al. designed 4,5,6,7-tetrahydrobenzo[d]thiazole-based DNA gyrase B inhibitors and incorporated these inhibitors with siderophore mimics. The siderophore mimic served as an inducer for increased uptake of the gyrase B inhibitors into the bacterial cytoplasm. Out of the ten gyrase B inhibitors tested against *P. aeruginosa*, four were able to inhibit ≥50% but only under iron-supplemented conditions, which is not reflective of the host environment during infection (Lamut et al., 2020). Several more attempts have been made at designing broad-spectrum anti-bacterial and anti-biofilm therapies targeting DNA gyrase or topoisomerase but none have shown good activity for *P. aeruginosa* (Dubey et al., 2012; Masih et al., 2020).

## LPS Modification

### Murepavadin

Murepavadin is a novel, non-lytic, species specific, outer-membrane protein targeting antibiotic for the treatment of *P. aeruginosa* infections, including those caused by MDR strains (Dale et al., 2018). Murepavadin is derived from the β-hairpin host defense molecule protegrin 1 (PG-1) and optimized to counteract unfavorable absorption, distribution, metabolism, excretion and toxicity (ADMET) properties normally associated with PG-1 (Obrecht et al., 2011). It is a macrocycle compound consisting of PG-1 loop sequences linked to a D-proline-L-proline sequence, the latter of which is important for its stability and subsequent strong antibacterial potential (Srinivas et al., 2010). Murepavadin functions through binding to the LPS transport protein D (LptD), an OMP necessary for LPS biogenesis in Gram-negative bacteria. The interaction between murepavadin and LptD causes inhibition of LPS transport, which leads to alterations of the LPS on the bacterial OM and eventually, cell death (Werneburg et al., 2012).

Murepavadin derivatives have been screened for activity against Gram-negative ESKAPE pathogens, including *P. aeruginosa*. These derivatives were initially shown to be effective, although the majority of leads were found to have high MIC values in the presence of 50% human serum and showed signs of lytic activity in human red blood cells. Therefore, compounds were generated consisting of β-hairpin macrocycles linked to the peptide macrocycle of polymyxin B. One of these compounds, compound 3, showed strong antimicrobial activity (MIC 0.5-2 μg/mL for *P. aeruginosa* isolates), low toxicity to mammalian cells, low plasma protein binding, good human plasma stability and no lytic activity towards human red blood cells. This compound was shown to perturb and permealise the bacterial membrane through interacting with the β-barrel domain of BamA in *E. coli* ATCC 25922 (Luther et al., 2019). BamA is part of the β-barrel assembly machinery BAM complex, which serves to fold and insert outer membrane proteins in the OM (Gu et al., 2016). The binding interaction between BamA and compound 3 locks BamA in its closed state through changing the conformational composite in the β-barrel lateral gate between open and closed states. It is not known what causes compound 3 to permeabilize the membrane. It may inhibit the folding activity of the BAM complex, leading to incorrectly folded proteins being misplaced in the inner membrane. Alternatively, BamA may only serve as an extra binding site for compound 3, thereby evading the LPS-modification resistance mechanism of Gram-negative pathogens (Luther et al., 2019).

## Quorum Sensing, Biofilm and Motility Attenuation

Quorum sensing regulates a wide range of genes involved in virulence and bacterial adaptation (Kalia, 2013). For instance, QS is required for the surfing and swarming motility phenotypes associated with increased resistance to antimicrobials. The surfing phenotype is regulated *via* three QS systems in *P. aeruginosa*; Las, Rhl and Pqs (Sun et al., 2018b). In addition, QS has been found to influence tolerance to antibiotics in *P. aeruginosa* biofilms. QS provides structural rigidity through the regulation of Pel polysaccharides and eDNA release necessary for the extracellular polysaccharide matrix. In addition, the production of rhamnolipids, surfactants important for the establishment and maintenance of biofilms, is controlled under QS (de Kievit, 2009). Therefore, QS has been recognized as a significant potential target for developing anti-resistance therapies. Strategies to combat antimicrobial resistance by targeting adaptive resistance mechanisms have significant potential for reversing antibiotic resistance in *P. aeruginosa*. Adaptive resistance is often mediated through complex global regulatory systems, such as the QS system, and regulate an extensive set of genes involved in resistance. Targeting these regulatory systems may prevent the activation of expression of these resistance genes that would normally be expressed under the environmental conditions of infection.

### Ajoene

Ajoene is a natural sulphur-containing compound extracted from garlic (Yoshida et al., 1998) that has been shown to modulate biofilm formation by inhibiting QS-induced production of virulence factors (Holm Jakobsen et al., 2012). It targets the Gac/Rsm component of QS, leading to a decreased expression of *rsmZ* and *rsmY* small regulatory RNAs. *rsmZ* and *rsmY* bind the global regulatory protein RsmA, and unbound RsmA represses the translation of genes by preventing ribosome binding to the Shine-Dalgarno site. Several genes involved in QS are under RsmA regulation, and low expression of *rsmZ* and *rsmY* in the presence of ajoene promotes RsmA mediated repression of these target genes (Jakobsen et al., 2017). However, the therapeutic applicability of ajoene is limited due to availability, instability, hydrophobicity and relatively high MIC values. Efforts are being undertaken to overcome these issues through modification, the use novel delivery systems and a targeted route of administration and through the development of synthetic ajoene analogues (Fong et al., 2017; Vadekeetil et al., 2019).

### Naringenin

Another novel QS-inhibitor derived from a natural source is the plant flavonoid naringenin. Naringenin diminishes the production of QS-regulated virulence factors in *P. aeruginosa* by binding directly to LasR, thereby competing with the activator of LasR, N-(3-oxo-dodecanoyl)-l-homoserine lactone (HSL).

It is ineffective at outcompeting HSL when the activator is already bound to LasR. Thus, the QS-inhibitor will only sufficiently interfere with the QS response when administered during early exponential growth, when naringenin can compete with unbound HSL for LasR binding. Naringenin is only suitable for combatting *P. aeruginosa* populations at low cellular densities, which often does not represent the clinical infection scenario. The full potential of QS-inhibition will only be realized if an inhibitor is developed that is capable of targeting *P. aeruginosa* QS signaling regardless of bacterial density and/or QS status (Hernando-Amado et al., 2020).

### Anti-Pseudomonal Drug Delivery Methods

Bacterial biofilms pose a physical barrier for drug penetration, which is one of the reasons that bacteria in a biofilm mode of growth are more resistant to antimicrobials. This phenomenon may be subverted with the use of nanocarriers that encapsulate antimicrobials and facilitate drug diffusion through the bacterial biofilm. In addition, nanocarriers can also protect drugs from degradation, ensure controlled drug release, and cause increased uptake by the drug target, leading to an overall higher efficiency of encapsulated drugs. Drug delivery methods can be diverse in chemical structure and nature (**Table 1**). Most published studies concur that encapsulated antibiotics are more effective at preventing or eradicating biofilm formation than their free drug counterpart (Alhariri et al., 2017; Shaaban et al., 2017; Zahra et al., 2017; Dai et al., 2018; Li et al., 2019), although several concluded that the activity of encapsulated and free antibiotic was equal (Severino et al., 2017; Wang et al., 2018; Hill et al., 2019).

**Table 1:** Recent efforts in the design of drug delivery methods for anti-pseudomonal therapies.

Chemical nature	Avr. Particle size (nm)	Cell type	Effect	
**Anionic liposome**	100	Planktonic	No difference between capsulated and free abx	Wang et al., 2018
	100	Biofilm	Enhanced biofilm eradication	Zahra et al., 2017
	625 - 806.6	Planktonic and biofilm	Prevents biofilm formation	Alhariri et al., 2017
**Graphene-oxide conjugates**	N/A	Biofilm	Enhanced biofilm eradication	Dai et al., 2018
**Poly(lactic-co-glycolic) acid nanoparticles**	229.4 - 469.2	Planktonic	No difference between capsulated and free abx	Hill et al., 2019
	132 - 348	Planktonic and biofilm	Prevents biofilm formation	Shaaban et al., 2017
**Solid lipid nanoparticles**	200 - 500	Planktonic	No difference between capsulated and free abx	Severino et al., 2017
**Water-soluble chitosan oligosaccharide conjugates**	N/A	Biofilm	Enhanced biofilm eradication	Li et al., 2019

### Nitric Oxide

Another promising antibiotic adjuvant targeting biofilms is the non-bactericidal, inhaled adjuvant, nitric oxide (NO). Exposure of *P. aeruginosa* biofilms to low-dose NO has been shown to cause dispersal of biofilms, rendering the infection susceptible to subsequent antibiotic treatment (Cai, 2020). NO functions by increasing bacterial phosphodiesterase activity which, in turn, leads to a reduction in the vital secondary signaling messenger, cyclic di-GMP. Cyclic di-GMP is vital for intracellular regulation of biofilm formation. Howlin et al. carried out *in vitro* biofilm studies using CF sputum clinical samples. Biofilms were treated with NO only, tobramycin only, tobramycin and ceftazidime, NO + tobramycin and NO + tobramycin and ceftazidime. Biofilms treated with NO showed a relative decrease in biofilm biomass and surface bound thickness in comparison to the untreated control. Further, treatment of biofilms with NO + tobramycin and NO + tobramycin and ceftazidime led to complete eradication of biofilm biomass and surface bound thickness. In comparison, biofilms treated with tobramycin or tobramycin and ceftazidime led to a 243% and 155% increase in biofilm biomass and a 199% and 174% increase in biofilm thickness, respectively, when compared to the untreated biofilm control. There is some evidence for the safety of NO administration in CF patients *in vivo* and NO is currently undergoing clinical trials to measure clinical efficacy (Howlin et al., 2017).

### PAAG (SNSP113)

Poly-acetyl-arginyl-glucosamine (PAAG), also called SNSP113, is a novel inhaled adjuvant therapy currently undergoing phase one clinical trials. PAAG is a polycationic glycoprotein that functions by permeabilizing the bacterial membrane and is active against methicillin resistant *Staphylococcus aureus* (MRSA), *Burkholderia* spp., *Mycobacterium* spp. and *E. coli*. PAAG has been shown to effectively disperse *Burkholderia cepacia* complex biofilm structures extracted from the CF lung (Narayanaswamy et al., 2019). In *P. aeruginosa*, PAAG is has been shown to effectively eradicate persister cells, which is important for the prevention of recurrent *P. aeruginosa* infections and subsequent exacerbations in people with CF (Narayanaswamy et al., 2018). In addition to serving as an effective antibiotic adjuvant, PAAG also reduces inflammation and promotes viscoelasticity and mucociliary clearance, making it a suitable drug candidate to improve the quality of life for patients with a variety of mucus diseases (Fernandez-Petty et al., 2019).

## Perspectives and Future Directions

The global overuse and misuse of antibiotics during the last 80 years has led to a profound increase in antimicrobial resistance. Between 2000 and 2010, global antibiotic consumption increased by nearly 70% and antibiotic resistant infections have accordingly become more pervasive, according to global epidemiological antimicrobial resistance surveillance networks (European Centre for Disease Prevention and Control, 2018) (Christaki et al., 2020). AMR is a complex, One Health issue, involving human, animal and environmental factors. The solution to AMR is therefore also likely to be a complex one, involving multiple strategies; maintaining AMR surveillance, containing AMR transmission, reducing selection pressure, developing novel antimicrobials or reverting antibiotic resistant microbes back to the susceptible phenotype with the use of antibiotic adjuvants (Hernando-Amado et al., 2019). Although progress in the development of naturally derived and peptide-based antimicrobials has been made (Mok et al., 2020; Upert et al., 2021). The conservation of existing antibiotics through careful stewardship is paramount to help mitigate the gap between the demand for new drugs and the diminishing supply pipeline. Antibiotic adjuvants will also play an important role in extending the shelf life of our existing antimicrobial therapeutic agents.

Adjuvant strategies targeting resistance mechanisms in *P. aeruginosa* could rejuvenate traditional antibiotic therapy by potentiating drug activity as well as slowing the development of antibiotic resistance. As described in this review, antimicrobial resistance in *P. aeruginosa* is regulated through a complex interplay of mechanisms. Resistance encoded in the core genome of *P. aeruginosa*, such as low outer membrane permeability, Mex-type efflux pumps and AmpC β-lactamase amount to the basal level of resistance against antimicrobials. This intrinsic resistance is present in the all *P. aeruginosa* strains and serves as a foundational level, which can be expanded upon. This expansion can be induced by environmental influences, such as host factors and signalling molecules, that switch on adaptive resistance mechanisms. Acquired resistance mechanisms, such as antibiotic target modifications generated *via* mutation, and antibiotic modifying enzymes or resistance plasmids, acquired by gene transfer, may serve as additional building blocks to expand the arsenal of resistance mechanisms a particular strain might carry. Several novel therapeutic strategies, targeting one or more of these mechanisms, have been described in this review. In light of recent findings, OM perturbants capable of sensitizing the Gram-negative bacterial membrane to previously non-active antibiotics seem an opportune strategy to combat resistance. OM perturbants can by-pass intrinsic as well as acquired and spontaneous resistance mechanisms, making them highly promising drug candidates, for which the development of resistance would be unlikely. However, efforts to finding perturbants suitable for targeting the *P. aeruginosa* membrane must be increased.

A second promising strategy is phage steering, which uses the natural predators of bacteria and the forces of evolutionary pressure to our advantage. Counterbalancing antibiotic resistance with phage susceptibility creates a double edged sword to circumvent key AMR mechanisms. In addition to these strategies, adjuvants targeting adaptive resistance mechanisms are worthy of consideration, due to the potential to disrupt multiple bacterial resistance and virulence processes with agents targeting a single regulator. Targeting global regulatory systems that would normally control the expression of resistance genes under infection conditions will prevent the activation of those genes with potential knock-on advantages in inhibition of virulence mechanisms. QS and two-component signaling systems are particularly attractive targets from this perspective, as are the regulators of biofilm formation.

There are several challenges in developing resistance-breaking therapy for *P. aeruginosa* infection. Firstly, due to its comparatively large genome and highly adaptive nature, a plethora of regulatory systems, as well as limited drug penetration and active efflux, many antibiotic adjuvants designed for Gram-negative pathogens do not show efficacy against *P. aeruginosa.* Secondly, toxicity has been proven to be the major hurdle for adjuvants designed against *P. aeruginosa*, leading to many being abandoned at early phases of development. Drug safety assessment is a long, expensive, but crucial process and toxicity is most likely where drug targets share structural similarity with human proteins. In this respect, bacterial signaling systems are good candidates, as prokaryotic and eukaryotic signaling systems are highly divergent, with eukaryotes lacking TCSS or phosphorelay systems.

As with all newly developed drugs designed to be used as combination therapy, care must be taken in determining the correct dosing and investigating clear synergy profiles. Drug levels necessary for synergy *in vitro* may not be achievable *in vivo.* Synergy *in vivo* may be affected by failure to obtain desired levels of drugs in the target tissue, drug metabolism or plasma protein binding. In addition, it is of paramount importance to evaluate drugs in relevant models that reflect the environmental conditions of infection. This will increase the predictive power of preclinical testing, reducing the costly progression of unpromising agents to clinical trials. The lack of well-validated *in vivo* models for testing CF anti-infective therapeutics limits the speed of development of new drugs. Murine models using cystic fibrosis transmembrane conductance regulator (CFTR) knockout animals, or transgenics in which the severe gut phenotype associated with loss of CFTR has been corrected are available, and have proved useful, but do not develop the characteristic features of acute and chronic *P. aeruginosa* infection seen in those with CF (Bragonzi, 2010; Semaniakou et al., 2019). Progress on the use of ferret and porcine infection models with mutated CFTR has been made, although these are limited by the availability of suitable immunobiology reagents (Keiser and Engelhardt, 2011). Intranasal administration of *P. aeruginosa* into the healthy murine lung often leads to either rapid clearance or sepsis. To create a model of persistent infection, it is usually necessary to immobilize *P. aeruginosa*. This can be achieved by encapsulating the bacteria into agar or alginate beads, where the bacteria are protected from clearance by immune effectors and where their mode of living more closely mimics bacterial biofilms present in chronic infection (Cigana et al., 2016). However, this bead model is technically demanding and requires surgical transtracheal instillation of the bead suspension, leading to additional complications and mortality not representative of bacterial infections in CF (Van Heeckeren and Schluchter, 2002). Alternatively, long term lung infection can be achieved using *P. aeruginosa* isolates from CF, some of which naturally establish chronic infection in mice, without the need for implantation into beads (Fothergill et al., 2014; Bricio-Moreno et al., 2018). This model has the advantages of using a natural infection route and having no requirement for surgical intervention, and offers opportunity to study lung infection over prolonged periods. However, the density of infection achieved in the lung is low, making some analyses challenging.

Questions remain regarding the commitment of governments and pharmaceutical manufacturers to ongoing investment in antibacterial drug development, particularly as financial and research priorities are reshuffled by the ongoing SARS-CoV2 crisis. Despite the understandable current emphasis on anti-viral agents and vaccines, it is important that we do not lose ground in the fight against AMR. Indeed, emerging evidence suggests that antibiotic use has increased dramatically in the COVID-19 era (Hsu, 2020). On top of this, increased usage of sanitizers and disinfectants globally may induce the development of cross-resistance to antibiotics. The ESKAPE pathogens, for which new medicines are urgently needed, continue to cause serious community-acquired and nosocomial infections, and if investment into research and drug development for these bacterial pathogens is diminished, it will exacerbate the global health and economic costs associated with the ongoing pandemic.

## Author Contributions

FL wrote the manuscript with supervision and input from all others. All authors contributed to the article and approved the submitted version.

## Funding

FL is supported by a PhD studentship from the Rosetrees Trust (M750). DN is supported by a Sir Henry Dale Fellowship funded by the Wellcome Trust and the Royal Society (Grant number 204457/Z/16/Z).

## Conflict of Interest

The authors declare that the research was conducted in the absence of any commercial or financial relationships that could be construed as a potential conflict of interest.
